# A systematic review and meta-analysis of asymptomatic malaria infection in pregnant women in Sub-Saharan Africa: A challenge for malaria elimination efforts

**DOI:** 10.1371/journal.pone.0248245

**Published:** 2021-04-01

**Authors:** Yonas Yimam, Mehdi Nateghpour, Mehdi Mohebali, Mohammad Javad Abbaszadeh Afshar

**Affiliations:** 1 Department of Medical Parasitology and Mycology, School of Public Health, Tehran University of Medical Sciences, Tehran, Iran; 2 Department of Biology, Faculty of Natural and Computational Sciences, Woldia University, Woldia, Ethiopia; 3 Centers for Research of Endemic Parasites of Iran (CREPI), Tehran University of Medical Sciences, Tehran, Iran; 4 Department of Medical Parasitology and Mycology, School of Medicine, Jiroft University of Medical Sciences, Jiroft, Iran; Institut de recherche pour le développement, FRANCE

## Abstract

**Background:**

In Sub-Saharan Africa (SSA), where malaria transmission is stable, malaria infection in pregnancy adversely affects pregnant women, fetuses, and newborns and is often asymptomatic. So far, a plethora of primary studies have been carried out on asymptomatic malaria infection in pregnant women in SSA. Nevertheless, no meta-analysis estimated the burden of asymptomatic malaria infection in pregnant women in SSA, so this meta-analysis was carried out to bridge this gap.

**Methods:**

PubMed, Web of Science, Scopus, Embase, and ProQuest were systematically searched for relevant studies published until 4 August 2020, and also the expansion of the search was performed by October 24, 2020. We assessed heterogeneity among included studies using I-squared statistics (I^2^). Publication bias was assessed by visual inspection of the funnel plot and further quantitatively validated by Egger’s and Begg’s tests. The pooled prevalence and pooled odds ratio (OR) and their corresponding 95% Confidence Interval (CI) were estimated using the random-effects model in Stata 15 software.

**Results:**

For this meta-analysis, we included 35 eligible studies. The overall prevalence estimate of asymptomatic *Plasmodium* infection prevalence was 26.1%% (95%CI: 21–31.2%, I^2^ = 99.0%). According to species-specific pooled prevalence estimate, *Plasmodium falciparum* was dominant species (22.1%, 95%CI: 17.1–27.2%, I^2^ = 98.6%), followed by *Plasmodium vivax*, *Plasmodium malariae* and *Plasmodium ovale*, respectively, found to be 3% (95%CI: 0–5%, I^2^ = 88.3%), 0.8% (95%CI: 0.3–0.13%, I^2^ = 60.5%), and 0.2% (95%CI: -0.01–0.5%, I^2^ = 31.5%). Asymptomatic malaria-infected pregnant women were 2.28 times more likely anemic (OR = 2.28, 95%CI: 1.66–3.13, I^2^ = 56.3%) than in non-infected pregnant women. Asymptomatic malaria infection was 1.54 times higher (OR = 1.54, 95%CI: 1.28–1.85, I^2^ = 11.5%) in primigravida women compared to multigravida women.

**Conclusion:**

In SSA, asymptomatic malaria infection in pregnant women is prevalent, and it is associated with an increased likelihood of anemia compared to non-infected pregnant women. Thus, screening of asymptomatic pregnant women for malaria and anemia should be included as part of antenatal care.

## Introduction

Malaria remains a substantial public health challenge that disproportionately affects SSA: in 2018, 213 million (93%) malaria cases worldwide and 405, 000 of all malaria-related deaths (94%) worldwide occurred in SSA [1]. *Plasmodium falciparum* is the etiologic agent for the vast majority of malaria cases in 2018 within the World Health Organization (WHO)-African Region [2]. Pregnant women are considered to be at higher risk of malaria and adversely affected by the disease [3]. In 2018, of 38, 971, 000 pregnant women, 11, 166, 000 pregnant women were diagnosed with malaria in SSA [1].

Clinical presentation of malaria during pregnancy vary depending on the intricate interplay of malaria transmission intensity in particular geographical settings, parasites, and the level of acquired immunity [3, 4]. In low malaria transmission areas, where pregnant women acquired low immunity, symptomatic malaria infection during pregnancy is commonly characterized by adverse consequences [5]. Nevertheless, in stable malaria transmission areas, where a considerable portion of the population are semi-immune, asymptomatic malaria infections in pregnant women are common [6, 7]. Asymptomatic malaria is the detection of *Plasmodium* species in peripheral blood, an axillary temperature <37.5°C, and an absence of malaria-related symptoms, given the lack of standard definition [8, 9]. In moderate and high malaria transmission areas, a significant proportion of asymptomatic malaria infections are caused by *P*. *falciparum*, while few infections are caused by other species of *Plasmodium* [10, 11].

In pregnancy, malaria affects not only pregnant women but also the fetus and newborn child [3, 12]. Malaria during pregnancy is characterized by sequestration of infected erythrocytes within the placenta with local inflammation and infiltration of immune cells (such as macrophages, monocyte, and lymphocytes) [13]. Such condition results in adverse consequence such as miscarriage, stillbirths, low birth weight, and infant mortality [12, 14, 15]. Asymptomatic malaria, if left untreated, can also progress to chronic infection leading to decreased erythropoiesis’ precursors, and increased erythrophagocytosis [6]. These changes contribute to maternal anemia that may lead to maternal mortality during pregnancy or post-partum, and it may result in increased infant/fetal mortality [16]. Moreover, asymptomatic malaria-infected individuals are a silent reservoir host of natural infection that usually goes undetectable and less likely to get treated, thus can transmit the disease to the population [17, 18].

In 2018, the WHO set a global goal for malaria elimination in at least 35 countries with malaria transmission in 2015 [2]. For the success of this campaign, intervention strategies that focused only on symptomatic infection are not adequate: thus, an additional intervention that also included asymptomatic individuals is of paramount importance [17–19]. Following progress from control to elimination of malaria in some countries, myriads of studies have been conducted on asymptomatic malaria infection in pregnant women in SSA [7, 20, 21]. A previous review (without meta-analysis) on asymptomatic plasmodial infection in pregnant women was conducted [22]. However, this review has not included meta-analysis and has not assessed factors associated with asymptomatic malaria infection. Additional studies have also been published since the publication of that review. We, therefore, conducted this systematic review and meta-analysis to synthesize pooled prevalence estimates of asymptomatic malaria and factors associated with asymptomatic malaria infection in pregnant women, which would be crucial for guiding future research and developing effective malaria elimination strategies.

## Methods

This systematic review and meta-analysis was carried out following Preferred Reporting Items for Systematic Reviews and Meta-analyses (PRISMA) guidelines (**S1 Checklist**).

### Data sources and searches

We conducted a systematic literature search using PubMed, Web of Science, Scopus, Embase, and ProQuest until 4 Aug 2020 to obtain studies that documented asymptomatic malaria infections in pregnant women in SSA. The search was performed in the English language; thus, research reports which were published other than the English language were not searched. Our search included the following terms: ’asymptomatic parasitemia’, ’asymptomatic malaria’, ’afebrile parasitaemia’, ’asymptomatic malaria*’, ’subclinical malaria’, ’asymptomatic plasmodium*’, ’subpatent malaria’, ’plasmodium vivax asymptomatic*’, ’plasmodium falciparum asymptomatic*’, ’afebrile malaria’, pregnan*, pregnancy, ‘pregnant women’, antenatal, ’prenatal care’, prenatal and ’antenatal care’. Search terms were used alone or in combination using Boolean operators (AND, OR). On October 24, 2020, we have expanded the search by including additional databases such as African Journal Online (AJOL), google scholar and open grey. The full description of the literature search approaches can be found in the **S1 File**. Also, we screened references of included studies and previous reviews on asymptomatic malaria infections for the inclusion of additional related studies that were not found by database search.

### Eligibility criteria

We applied the PICOS (P: population, I: intervention, C: comparators, O: outcome, S: study design) approach to include eligible studies. (i) P: healthy pregnant women with no symptoms/signs suggestive of malaria usually fever (axillary temperature <37.5°C). (ii) I: none (iii) C: those pregnant women who have been tested negative for asymptomatic malaria infection, or groups (i.e. anemic Vs non-anemic, primigravida (first pregnancy) Vs multigravida (at least two previous pregnancy), and first and second trimester Vs third trimester). (iv) O: asymptomatic malaria infection is characterized by the detection of Plasmodium infection in the absence of clinical symptoms of malaria. The detection of Plasmodium infection can be performed using rapid diagnosis methods, microscopy or molecular methods, or in combination. And anemia is characterized as a level of hemoglobin < 11 g/dl [23] (v) S: observational studies that reported asymptomatic malaria infections or asymptomatic malaria infection prevalence can be calculated from available data. We excluded studies according to the following reasons: (i) studies evaluating symptomatic malaria infections in pregnant women, but when both symptomatic and asymptomatic malaria infections were reported, we extracted the data exclusively related to asymptomatic malaria infection (ii) studies evaluating asymptomatic malaria infections but conducted outside of SSA, (iii) non-primary studies such as reviews and case reports.

### Study selection and data extraction

All studies which were retrieved using databases and manual hand searching, potentially relevant for inclusion, were stored in EndNote X 8 for management. First, duplicate studies were automatically excluded, and then titles and abstracts of the remaining studies were screened. Second, we have removed titles and abstracts which appear irrelevant to inclusion. Third, after deduplication and screening of titles and abstracts, potentially relevant studies have been scrutinized for full-text (where available) and ineligible studies were removed. Fourth, literature in the full-texts were filtered based on pre-specified criteria.

After the selection of eligible studies, the following data were extracted using a pre-prepared excel sheet: Author/s and year of publication, country, diagnostic methods, number of pregnant women tested for asymptomatic malaria infection, number of asymptomatic malaria positive study participants. Where the data is available, the following data were also captured; gravidity, gestational age (first, second, and third trimester), and hemoglobin status. Two authors (YY and MJA) independently conducted study selection and data extraction. And any inconsistency or disagreement between the two authors was resolved by consensus.

### Quality assessment

Two authors (YY and MJA) independently assessed the quality of included studies using a Hoy et al. [24], risk of a bias assessment tool for cross-sectional studies. This tool has ten items aimed at evaluating internal and external validity. Six items: data collection, appropriateness of case definition, reliability and validity of study instrument, method of data collection, duration of prevalence period, and correctness of numerator and denominator were used to investigate the internal quality, while four items: representation, sampling frame, random selection, and, non-response bias were used to examine the external validity of included studies. Every single item was scored with a low or moderate or high risk of bias. And the unclear risk of bias was taken as a high risk of bias. The quality of each study based on high risk of bias was rated as low, moderate, and the high risk by summary score ≥2, 3–4, and ≥5, respectively (**S2 File**).

### Data analysis

All statistical analyses were carried out using *metan* written command in Stata software (version 15). To synthesize the pooled odds ratios (ORs), we used 2 x 2 tables to extract data (**S3 File**). When there were zero cases, we added 0.5 as a contingency correction. ORs and 95% Confidence Interval (CI) were calculated to examine the relationship between asymptomatic malaria infections and anemia, gravidity, and gestational age. The overall pooled prevalence and pooled ORs estimate were computed using the DerSimonian-Laird method for the random-effects model, based on the inverse variance approach for measuring weight. Inconsistency index (I^2^ statistics) was used to assess the magnitude of heterogeneity among included studies, with I^2^ value >25%, 25–75%, <75% were taken as low, moderate, and high heterogeneity, respectively [25]. We assessed publication bias by visual inspection of the funnel plot and further validated using Egger’s regression test [26] and Begg’s correlation test [27]. We did a subgroup analysis based on diagnostic methods and the risk of bias to assess the potential source of heterogeneity between studies. We have conducted a univariate meta-regression analysis based on publication years of eligible studies and sample size. To assess the overall pooled estimate was not impacted by a single study, we did a one study leave-out sensitivity analysis.

## Results

**Fig 1:**
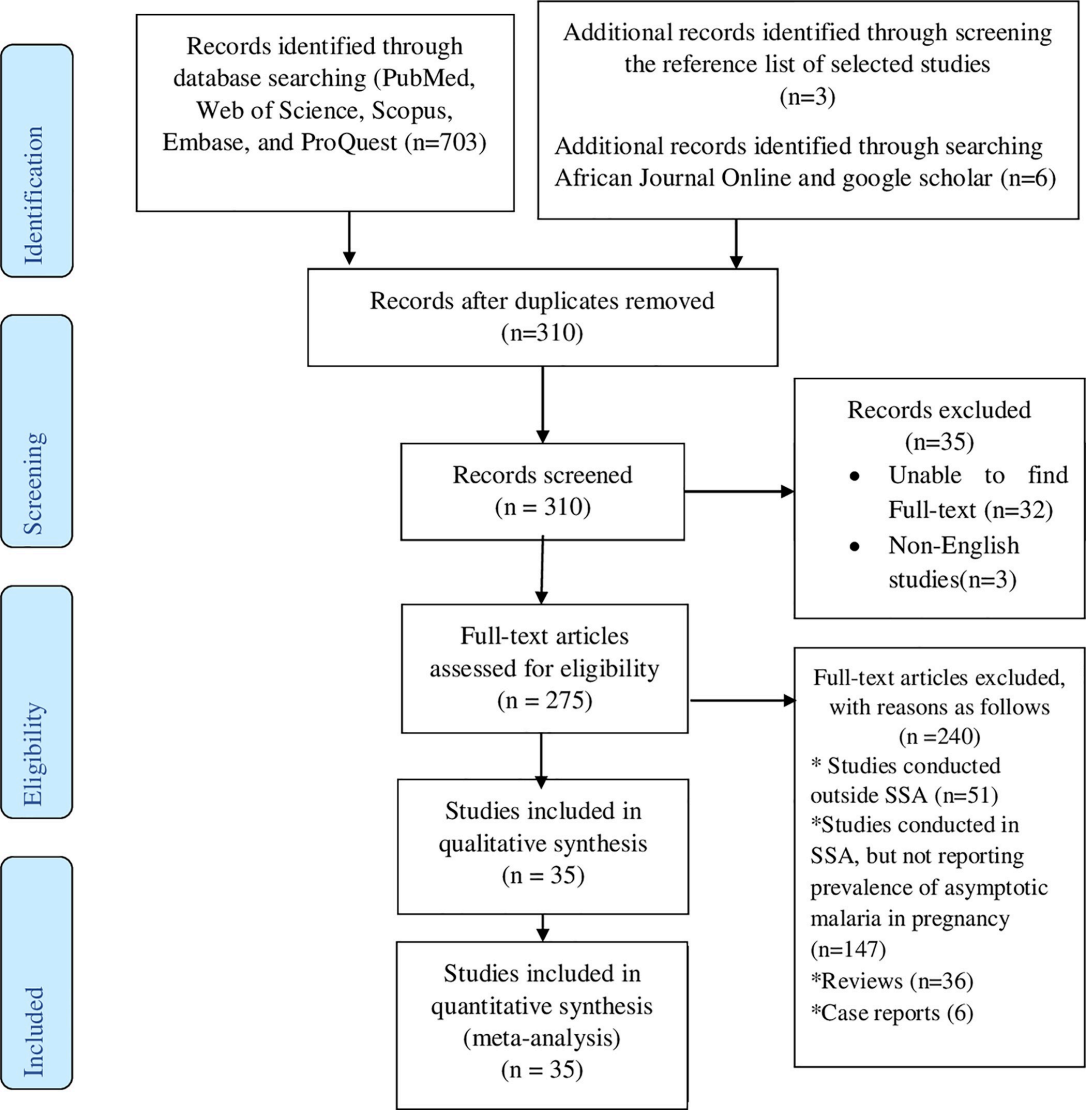
Flow diagram showing studies selection process implemented for this meta-analysis.

**Fig 1** shows electronic data sources and studies selection process. Initially, a total of 703 records were identified from PubMed, Web of Science, Scopus, Embase, and ProQuest. After removing 393 duplicate records, 310 remaining studies were screened based on titles and abstracts and 35 records were further excluded because we were unable to find full-texts. Of 35 studies, 32 records titles and abstracts were not related to the objectives of this study, while 3 potentially relevant reorders were published other than English language. The remaining 275 studies were screened by critically reading the full-texts. Of the 275 full-text studies, we excluded 240 studies with reasons: 51 of them were conducted outside of SSA; 147 of them did not report the prevalence of asymptotic malaria in pregnancy despite studies were conducted in SSA; 36 of them were reviews, and 6 of them were case reports. Twenty-six studies were deemed eligible for inclusion and three additional relevant studies were found from the references list of included studies that were not identified by our electronic database search strategies. Also, additional six eligible studies were identified through searching African Journal Online and google scholar. In the end, a total of 35 studies were included in this meta-analysis.

### Characteristics of included studies

The characteristics of 35 eligible studies included in this systematic review and meta-analysis is presented in **S1 Table**. All included studies were conducted in fourteens different countries; the largest number of included studies (n = 16, 45.7%) was conducted in Nigeria, while three in Ethiopia, two in DR Congo, two in Burkina Faso, two in Tanzania, one in Ghana, one in Kenya, one in Malawi, one in Madagascar, one in Liberia, one in Uganda, one in one in Zambia, one in Mozambique, one in Gabon, and one in Cameroon. The largest number of included studies (n = 20) used microscopy to diagnose asymptomatic malaria, while the remaining nine, four, and two used molecular methods, microscopy and rapid diagnostic test, and rapid diagnostic test, respectively. The sample size of eligible studies ranges from 50 to 2459. All included studies were conducted between 2002 and 2020, with 9, 7, and 18 of the included studies were conducted between 2002 and 2010, 2011–2014, and 2015–2020, respectively. The risk of bias assessment of included studies showed that, respectively, 17 and 18 of them had a low and moderate risk of bias.

### Publication bias assessment

We evaluated small-studies effects (publication bias) on our pooled prevalence estimates by visual inspection of the funnel plot. According to the qualitative evaluation of funnel plot symmetry, it seems there is no evidence of publication bias (**S1 Fig**). To quantitatively confirm the result of the funnel plot, we performed Egger’s regression test and Begg’s correlation test. Both Egger’s regression test (P = 0.287) and Begg’s correlation test (P = 0.966) showed the absence of publication bias. Additionally, we performed the trim and fill method and there were no filled studies that pinpoint the lack of detectable publication bias. We have not assessed publication bias on pooled ORs estimates because of the limited number of included studies.

### The overall prevalence of asymptomatic malaria infection in pregnant women in SSA

**Fig 2:**
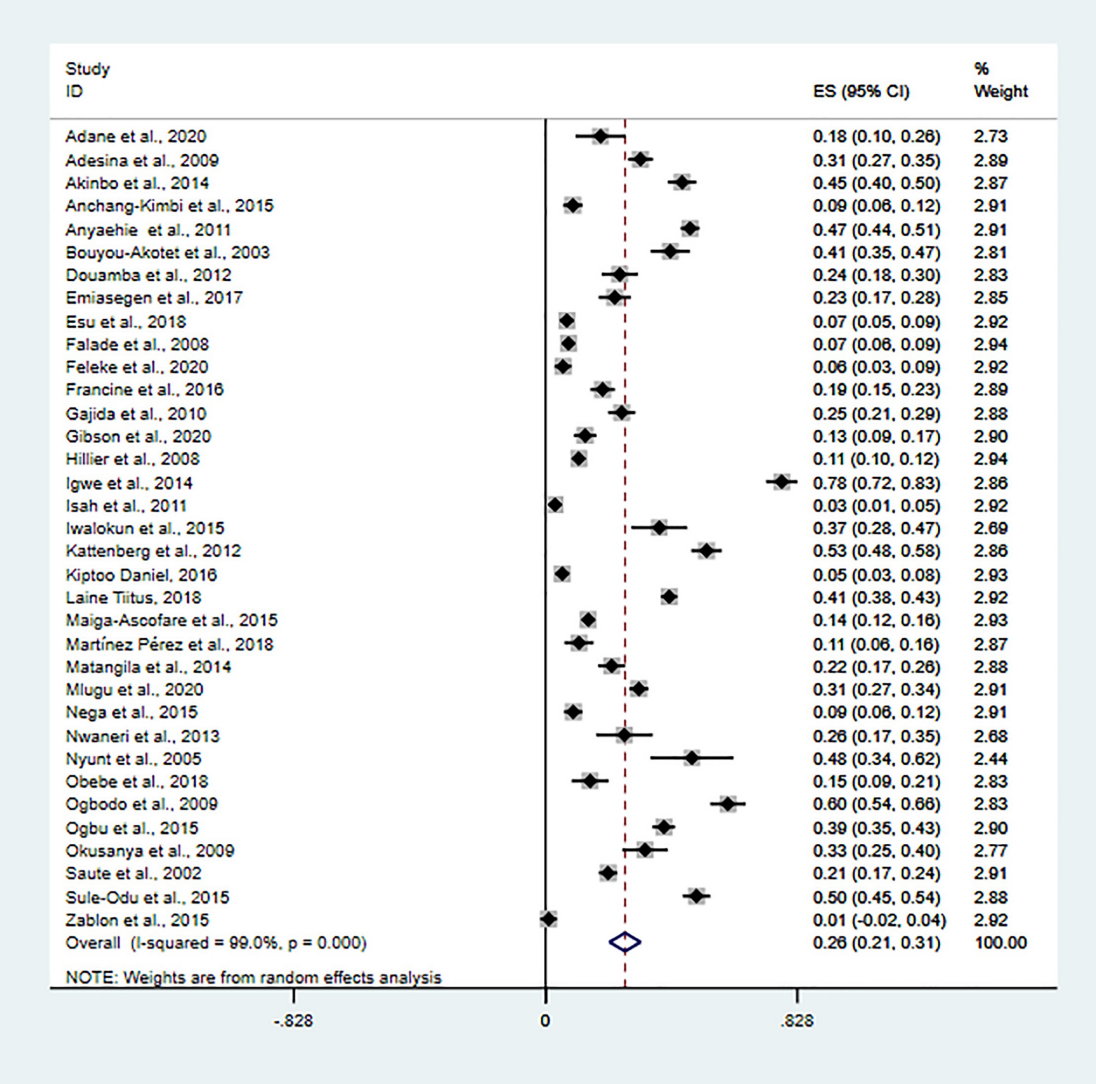
The overall prevalence of asymptomatic malaria infection.

The overall prevalence of asymptomatic malaria infection was 26.1% (95%CI: 21–31.2%) using a random-effects model, with very high heterogeneity (I^2^ = 99%, P<0.01) **(Fig 2)**. Of the thirty five included studies, twenty-seven had reported prevalence of species-specific asymptomatic malaria infection. According to species-specific pooled prevalence estimate (number of *P*. *falciparum*/*P*. *malariae*/*P*. *falciparum* /*P*. *vivax* positive divided by total number of tested), *P*. *falciparum* was dominant species (22.1%, 95%CI: 17.1–27.2%, I^2^ = 98.6%, P<0.01, n = 27), followed by *P*. *vivax*, *P*. *malariae* and *P*. *ovale*, respectively, found to be 3% (95%CI: 0–5%, I^2^ = 88.3%, P<0.01, n = 4), 0.8% (95%CI: 0.3–0.13%, I^2^ = 60.5%, P<0.01, n = 4), and 0.2% (95%CI: -0.1–0.5%, I^2^ = 31.5%, P<0.01, n = 3). Mixed infection pooled prevalence estimate of *P*. *falciparum* and *P*. *malariae*, *P*. *falciparum* and *P*. *vivax*, and *P*. *falciparum* and *P*. *ovale* was (2%, 95%CI: -1-5%, I^2^ = 95.2%, P<0.01, n = 2), (1%, 95%CI: 0–2%, I^2^ = 0.0%, P<0.01, n = 2), and (0.3%, 95%CI: -0.2–0.8%, I^2^ = 71.3%, P<0.01, n = 2), respectively.

Overall pooled prevalence estimate of asymptomatic malaria infection was significantly higher in studies that diagnosed asymptomatic malaria using molecular methods (30%, 95%CI: 20–40%, I^2^ = 98.2%, P<0.01, n = 9) and microscopy (28%, 95%CI: 21–35%, I^2^ = 99.2%, P<0.01, n = 20), compared to studies that used microscopy and rapid diagnostic method (12%, 95%CI:3–21%, I^2^ = 94.9%, P<0.01, n = 4) and rapid diagnostic method (14%, 95%CI: -8-36%, I^2^ = 95.4%, P<0.01, n = 2). Subgroup analysis of pooled prevalence estimate stratified by risk bias assessment was (24%, 95%CI: 16–32%, I^2^ = 98.8%, P<0.01, n = 16) and (28%, 95%CI: 21–35%, I^2^ = 99.0%, P<0.01, n = 19) for low and moderate risk of bias, respectively. Also, subgroup analysis stratified by region was 19% (95%CI: 12–25%, I^2^ = 98.5%, P<0.01, n = 11), 31% (95%CI: 22–40%, I^2^ = 99.2%, P<0.01, n = 20), and 22% (95%CI: 11–34%, I^2^ = 96.4%, P<0.01, n = 4) in Eastern, Western and Central African countries.

### Pooled odds ratio estimate

**Fig 3:**
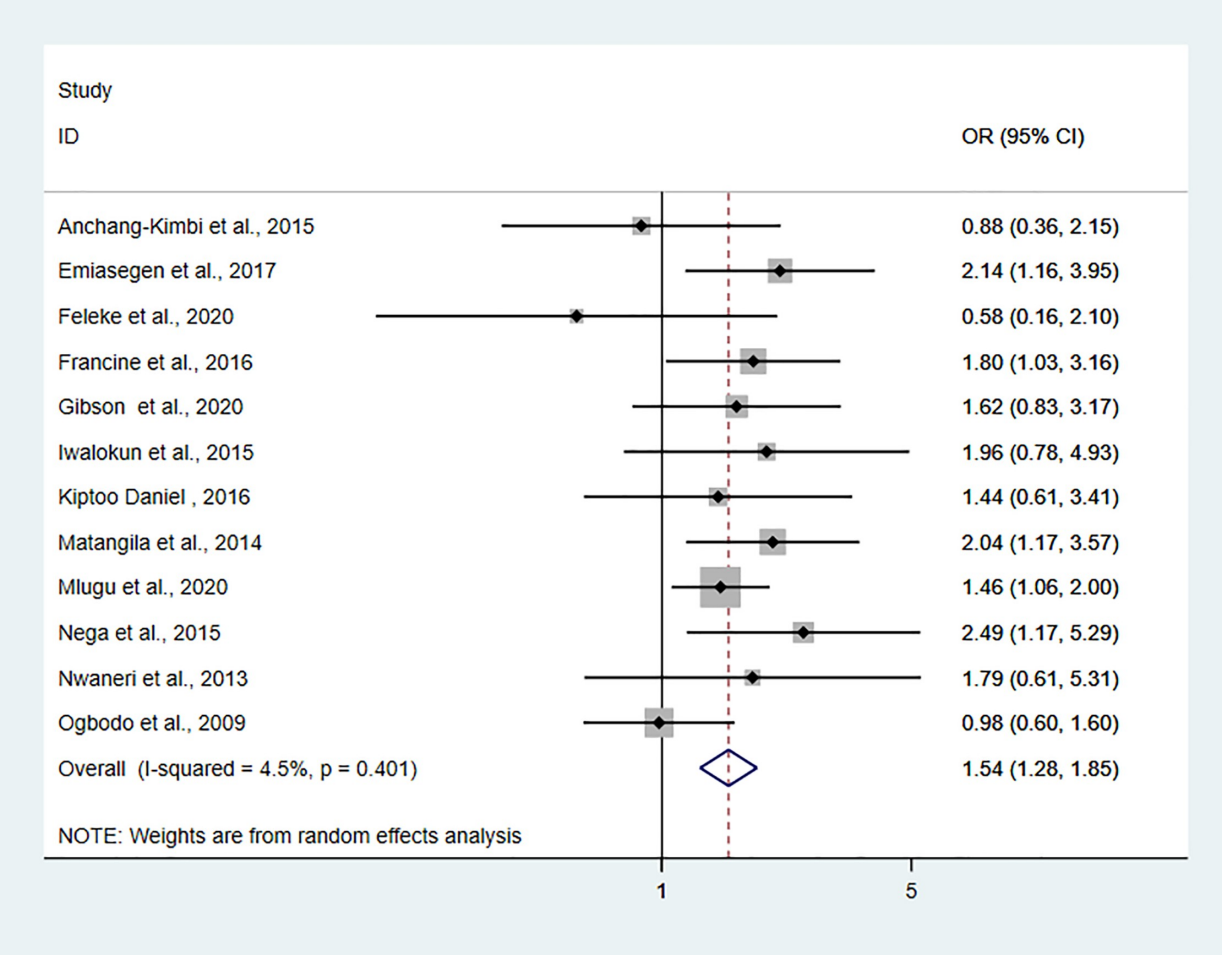
The pooled odds ratio of asymptomatic malaria infection in primigravidae women compared to multigravida women.

**Fig 4:**
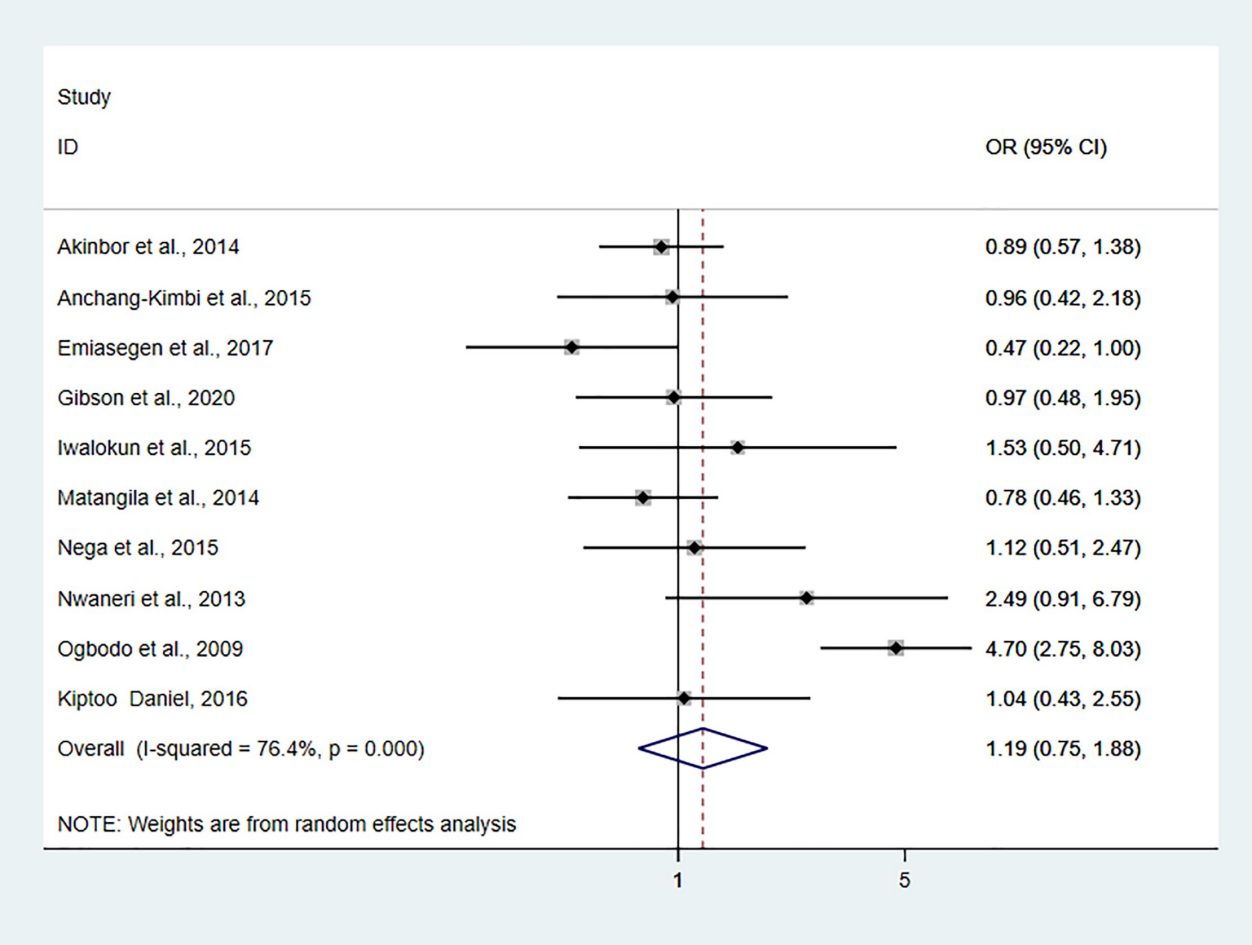
Forest plot showing pooled odds of asymptomatic malaria infection in the first and second trimester compared to third-trimester pregnant women.

We included nine studies to explore the relationship between asymptomatic malaria infection and anemia in pregnant women. The pooled odds ratio estimate (OR = 2.28, 95%CI: 1.66–3.13, I^2^ = 56.3%, P = 0.019) showed that the odds of anemia in pregnant women infected with asymptomatic malaria infection was 2.28 higher than in pregnant women not infected with asymptomatic malaria (**S2 Fig**). Pooled odds of asymptomatic malaria infection in primigravidae women was 1.55 times higher (OR = 1.54, 95%CI: 1.28–1.85, I^2^ = 4.5%, P = 0.401) compared to multigravida pregnant women (**Fig 3**). Pooled odds of asymptomatic malaria infection in the first and second trimester was 1.24 times higher than pregnant women in the third trimester, albeit statistically not significant (OR = 1.19, 95%CI: 0.75–1.88, I^2^ = 76.4%, P<0.001) (**Fig 4**).

### Meta-regression analysis

We carried out a meta-regression analysis to explore the trends of asymptomatic malaria infection in pregnant women over the past two decades. The result of the meta-regression analysis pointed out that the overall prevalence of asymptomatic malaria infection is not showed a statistically significant rise between the years 2002 and 2020(Coef. -.0221807, P = 0.200) (**S3 Fig**). Also, the overall estimate of asymptomatic malaria infection was not impacted by an increase in sample size (Coef. -.0000877, P = 0.55) (**S4 Fig**).

### Sensitivity analysis

We performed a sensitivity analysis to assess the robustness of our pooled prevalence estimate. The result of sensitivity analysis demonstrated that the overall estimate of asymptomatic malaria infection prevalence was robust and it was not influenced by a single study. The highest and lowest overall prevalence of asymptomatic malaria infection was 26.79% (95%CI: 21.54–32.04%) [28] and 24.55% (95%CI: 19.76–29.33%) [29], respectively, when a single study omitted and it is similar to the pooled overall prevalence estimate (26.1% (95%CI: 21–31.2%) (**S5 Fig**).

## Discussion

Asymptomatic malaria infection is a challenge for malaria elimination campaigns in such a way that asymptomatic individuals do not show overt clinical symptoms, they are less likely to seek health service, so they become a silent natural reservoir of infection that facilitates sustainable malaria transmission [17, 18]. Particularly, in pregnancy, asymptomatic malaria infection has adverse consequences on the maternal, fetus, and new-borns health [3, 8, 14]. WHO recommends measures to curb malaria during pregnancy, including consistent use of insecticide‐treated net and intermittent preventive treatment with sulfadoxine‐pyrimethamine during the second and third trimesters with at least one month apart [30, 31]. The limitations of the latter measure are the gap in the first trimester as the pregnant women are often received late for their first antenatal care visit in Africa and resistance to sulfadoxine–pyrimethamine treatment in East Africa and southern Africa [32]. Although a previous review (with no meta-analysis) assessed the global scenario of asymptomatic plasmodial infection in pregnant women [22], a meta-analysis of asymptomatic malaria infection in pregnant women in SSA has not been performed to date, so this systematic with subsequent meta-analysis was carried out. In this study, a total of 35 studies involving 17, 149 pregnant women were used to provide an overall prevalence of asymptomatic malaria infection in SSA. Thus, this study may provide useful and valuable information for guiding future research and developing effective malaria elimination strategies.

This study demonstrated that asymptomatic malaria infection was prevalent in SSA, with an overall pooled prevalence of 26.1%. The overall prevalence of asymptomatic malaria infections in the present study was significantly higher than studies conducted in low malaria transmission areas including Thailand (11.4%) [33] and Bangladesh (2.3%) [34]. However, the result of this meta-analysis is relatively similar to studies that carried out in moderate to high malaria transmission areas such as Burkina Faso (24%) [35] and DR Congo (29%) [6]. This highest prevalence of asymptomatic malaria infection in moderate to high malaria transmission settings might be because of acquired immunity due to exposure [4]. Individuals residing in moderate to high malaria transmission areas are frequently exposed to *Plasmodium* infections over time which leads to the development of partial anti-disease immunity [4]. However, this immune response is not sterile and it might be lost in the absence of repeated exposure to the parasite [36, 37]. Subgroup analysis stratified by diagnostic methods showed an overall prevalence of 30%, 28%, and 14% using the molecular method, microscopy, and rapid diagnostic methods. The molecular method has demonstrated a higher overall prevalence compared to the rapid diagnostic method and microscopy. This might be related to the high sensitivity of molecular methods that allow the detection of 0.5–5 parasites per μL of blood, while microscopy and RDT are unlikely to detect parasite densities less than 100 parasites/μL of blood which make them insufficiently sensitive for detection of malaria in asymptomatic and submicroscopic patients [38, 39]. This underscores the importance of better sensitive PCR-based tools to detect low levels of parasitaemia in asymptomatic patients relevant to malaria elimination [39].

Among twenty-seven included studies that reported species-specific asymptomatic malaria infection, *P*. *falciparum* was the predominant species with an overall prevalence of 22.1% while *P*. *vivax*, *P*. *malariae*, and *P*. *ovale* overall prevalence was 3%, 0.8%, and 0.2%, respectively. This result is in agreement with the WHO 2018 report that indicated *P*. *falciparum* accounted for 99.7% of malaria in the WHO African Region [1].

In this study, the result of the pooled odds ratio revealed that asymptomatic malaria-infected pregnant women were 2.28 times more likely to be anemic than pregnant women who were not infected with asymptomatic malaria infection. This result is comparatively similar to primary studies conducted in Ethiopia [40] and DR Congo [6] that indicated asymptomatic malaria-infected pregnant women were 3.78 and 5 times more likely anemic than non-infected pregnant women, respectively. The complete mechanisms of anemia related to malaria are not fully understood: it is suggested that malaria infection causes destruction of red blood cells (erythrophagocytosis) as well as disturbance of red blood cell formation (dyserythropoiesis) that eventually contribute to maternal anemia [41, 42]. However, the relationship between malaria and anemia is complex and controversial: iron deficiency can reduce infection with malaria, but malaria infection, in turn, is associated with anemia at the level of both individuals and populations [43].

The finding of this study showed that the prevalence of asymptomatic malaria infection was 1.54 times more likely higher in primigravida women compared to multigravida women. Studies conducted in stable malaria transmission areas have also shown that asymptomatic malaria infection is reduced in increasing gravidity [3, 44]. The decreased in the occurrence of asymptomatic malaria infection with increased gravidity may be related to exposure-acquired immunity. This could be explained by the fact that asymptomatic malaria infection of *P*. *falciparum* results in sequestration and adherence of infected red blood cells with chondroitin sulfate A (CSA) on the surface of the placenta, which is mediated by parasite-encoded variant surface antigens (VSA) [45]. Anti-adhesion antibodies against CSA-binding parasites limit parasite accumulation and contribute to a reduction in the prevalence of malaria [45]. Since primigravida women are less exposed to malaria infection compared to multigravida, they lack anti-adhesion antibodies, thus primigravida women are more susceptible to malaria infection compared to multigravida women [45, 46].

In this study, some limitations are noticed. First, we performed a literature search using the English language, and we excluded three studies published other than the English language. This might not significantly impact the result although language bias was difficult to be avoided. Second, studies included in this meta-analysis are not from all African countries. Taking into account the expected variation across countries, the findings of this study might not be representative of the whole African countries. Third, a high level of heterogeneity was observed among studies that could be related to heterogeneity in diagnostic methods and publication years of studies. Given these aforementioned inherent limitations, the result of this study should be interpreted with caution.

## Conclusion

The findings of the present study demonstrate that asymptomatic malaria infection is prevalent in pregnant women in SSA. *P*. *falciparum*, *P*. *vivax*, *P*. *ovale*, and *P*. *malariae* were detected from asymptomatic malaria-infected pregnant women: However, *P*. *falciparum* was the predominant causative agent of asymptomatic malaria. Again, the prevalence of anemia in pregnant women infected with asymptomatic malaria was higher than in non-infected pregnant women. Also, primigravidae women were more likely infected with asymptomatic malaria infection compared to multigravida pregnant women. Therefore, laboratory diagnosis of asymptomatic pregnant women for malaria and anemia should be included as part of antenatal care follow-up. Also, further large-scale studies using a highly sensitive method should be conducted to better understand the burden of asymptomatic malaria in pregnancy in SSA.

## Supporting information

S1 Checklist PRISMA guideline. (DOC)

S1 Table Characteristics of studies included in this meta-analysis. (DOCX)

S1 File Search strategy. (DOCX)

S2 File Quality assessment of sleeted studies. (DOCX)

S3 File Data extraction for pooled odds ratio estimate. (DOCX)

S1 Fig Funnel plot of the arcsine transformed prevalence estimates (t) of asymptomatic malaria infection in Sub-Saharan Africans, 2002 to 2020. Abbreviation: se of t, standard error of t.
(TIF)

S2 Fig Forest plot showing the relationship between asymptomatic malaria infection and anemia in pregnant women in Sub-Sharan African. (TIF)

S3 Fig Meta-regression analysis showing trends of asymptomatic malaria infection. (TIF)

S4 Fig Forest plot depicting meta-regression analysis of asymptomatic malaria infection based on the sample size. (TIF)

S5 Fig Forest plot showing sensitivity analysis. (TIF)
