# Supplementary material for: A systematic review and meta-analysis of asymptomatic malaria infection in pregnant women in Sub-Saharan Africa: A challenge for malaria elimination efforts
Source: PLoS One. 2021 Apr 1;16(4):e0248245. doi: 10.1371/journal.pone.0248245 (PMC8016273; doi:10.1371/journal.pone.0248245)
Supplement: S1 Table — (DOCX) [file pone.0248245.s002.docx]

S1 Table: characteristics of studies included in this meta-analysis

| Study  Ref.  No. | Author/s and year | Country | No.  Positive | No.  Tested | *Plasmodium* species (No. positive) | Diagnostic method | Risk of bias assessment |
| --- | --- | --- | --- | --- | --- | --- | --- |
| [1] | Adane et al., 2020 | Ethiopia | 15 | 83 | Pf=8, Pv=5, Pf+Pv=2 | Mo | Low |
| [2] | Adesina et al., 2009 | Nigeria | 156 | 500 | Pf=156 | Mi | Moderate |
| [3] | Akinbo et al., 2014 | Nigeria | 197 | 439 | NR | Mi | Moderate |
| [4] | Anchang-Kimbi et al., 2015 | Cameroon | 27 | 301 | Pf=27 | Mi | Moderate |
| [5] | Anyaehie et al., 2011 | Nigeria | 470 | 990 | NR | Mi | Low |
| [6] | Bouyou-Akotet et al., 2003 | Gabon | 93 | 227 | Pf=93 | Mi | Moderate |
| [7] | Douamba et al., 2012 | Burkina Faso | 49 | 201 | Pf=49 | Mi and RDT | Moderate |
| [8] | Emiasegen et al., 2017 | Nigeria | 55 | 242 | Pf=55 | Mi | Low |
| [9] | Esu et al., 2018 | Nigeria | 32 | 459 | Pf=32 | Mi | Low |
| [10] | Falade et al., 2008 | Nigeria | 138 | 1848 | NR | Mi | Moderate |
| [11] | Feleke et al., 2020 | Ethiopia | 15 | 263 | Pf=9, Pv=6 | Mi | Low |
| [12] | Francine et al., 2016 | DR Congo | 68 | 363 | Pf=68 | Mi | Low |
| [13] | Gajida et al., 2010 | Nigeria | 90 | 360 | NR | Mi | Low |
| [14] | Gibson et al., 2020 | Kenya | 40 | 308 | Pf=35, Pm=3, Po=2 | Mi | Moderate |
| [15] | Hillier et al., 2008 | Uganda | 268 | 2459 | Pf=268 | Mi | Moderate |
| [16] | Igwe et al., 2014 | Nigeria | 194 | 250 | Pf=194 | Mi | Moderate |
| [17] | Isah et al., 2011 | Nigeria | 7 | 225 | Pf=7 | RDT | Low |
| [18] | Iwalokun et al., 2015 | Nigeria | 40 | 107 | Pf=40 | Mo | Low |
| [19] | Kattenberg et al., 2012 | Burkina Faso | 201 | 380 | Pf=201 | Mo | Low |
| [20] | Kiptoo Daniel, 2016 | Ghana | 22 | 400 | Pf=21, Pm=1 | Mi | Moderate |
| [21] | Laine Tiitus, 2018 | Malawi | 531 | 1308 | NR | Mo | Low |
| [22] | Maiga-Ascofare et al., 2015 | Madagascar | 174 | 1242 | Pf=157, Pv=1, Po=1, Pm=9, Pf+Pm=5, Pf+Po=1 | Mo | Moderate |
| [23] | Martínez Pérez et al., 2018 | Liberia | 18 | 164 | Pf=18 | Mo | Moderate |
| [24] | Matangila et al., 2014 | DR Congo | 72 | 332 | Pf=72 | Mo | Low |
| [25] | Mlugu et al., 2020 | Tanzania | 251 | 819 | Pf=202, Po=3, Pm=13, Pf+Pm=28, Pf+Po=5 | Mo | Low |
| [26] | Nega et l. 2015 | Ethiopia | 31 | 341 | Pf=12, Pv=15, Pf+ Pv=4 | Mi and RDT | Low |
| [27] | Nwaneri et al., 2013 | Nigeria | 22 | 85 | Pf=22 | RDT | Moderate |
| [28] | Nyunt et al., 2005 | Zambia | 25 | 52 | Pf=25 | Mo | Low |
| [29] | Obebe et al., 2018 | Nigeria | 19 | 130 | Pf=19 | Mi and RDT | Low |
| [30] | Ogbodo et al., 2009 | Nigeria | 163 | 272 | Pf=163 | Mi | Low |
| [31] | Ogbu et al., 2015 | Nigeria | 256 | 659 | NR | Mi | Moderate |
| [32] | Okusanya et al., 2009 | Nigeria | 49 | 150 | NR | Mi | Moderate |
| [33] | Saute et al., 2002 | Mozambique | 138 | 672 | Pf=138 | Mi | Low |
| [34] | Sule-Odu et al., 2015 | Nigeria | 232 | 468 | NR | Mi | Moderate |
| [35] | Zablon et al., 2015 | Tanzania | 0 | 50 | Pf=0 | Mi and RDT | Moderate |

Note: Pf ; *Plasmodium falciparum*, Pv: *Plasmodium vivax*, Po: *Plasmodium ovale*, Pm= *P. malariae*, Pf+Pm: *P. falciparum* and *P. malariae* mixed infection, Pf+Po= *P. falciparum* and *P. ovale* mixed infection, NR; Not reported, Mi; Microscopic examination, Mo; Molecular method Mi and RDT; Microscopic examination and Rapid Diagnostic Test, RDT; Rapid Diagnostic Test

References

1. Adane T, Yimer M, Gelaye W, Tegegne B. Prevalence of asymptomatic Plasmodium species infection and associated factors among pregnant women attending antenatal care at Fendeka town health facilities, Jawi District, North west Ethiopia: A cross-sectional study. PLoS One. 2020;15(4):1-3.

2. Adesina KT, Balogun OR, Babatunde AS, Sanni MA, Fadeyi A, Aderibigbe S. Impact of malaria parasitaemia on haematologic parameters in pregnant women at booking in Ilorin, Nigeria. Trends in Medical Research. 2009;4(4):84-90.

3. Akinbo FO, Osanyinbi B, Omoregie R, Ande ABA. Asyptomatic malaria among pregnant women in Edo State, Nigeria. Journal of Medicine and Biomedical Research. 2014;13(1):61-9.

4. Anchang-Kimbi JK, Nkweti VN, Ntonifor HN, Apinjoh TO, Tata RB, Chi HF, et al. Plasmodium falciparum parasitaemia and malaria among pregnant women at first clinic visit in the mount Cameroon Area. BMC infectious diseases. 2015;15(1):1-10.

5. Anyaehie U, Nwagha UI, Aniebue PN, Nwagha TU. The effect of free distribution of insecticide-treated nets on asymptomatic Plasmodium parasitemia in pregnant and nursing mothers in a rural Nigerian community. Nigerian journal of clinical practice. 2011;14(1):19-22.

6. Bouyou-Akotet MK, Ionete-Collard DE, Mabika-Manfoumbi M, Kendjo E, Matsiegui P-B, Mavoungou E, et al. Prevalence of Plasmodium falciparum infection in pregnant women in Gabon. Malaria journal. 2003;2(1):1-7.

7. Douamba Z, Bisseye C, Djigma FW, Compaoré TR, Bazie VJ, Pietra V, et al. Asymptomatic malaria correlates with anaemia in pregnant women at Ouagadougou, Burkina Faso. Journal of biomedicine & biotechnology. 2012;2012:1-6.

8. Emiasegen SE, Giwa FJ, Ajumobi O, Ajayi I, Ahmed SA, Olayinka AT. Asymptomatic Plasmodium falciparum parasitaemia among pregnant women: a health facility-based survey in Nassarawa-Eggon, Nigeria. Malaria World J. 2017;15(24):25-34.

9. Esu E, Tacoli C, Gai P, Berens-Riha N, Pritsch M, Loescher T, et al. Prevalence of the Pfdhfr and Pfdhps mutations among asymptomatic pregnant women in Southeast Nigeria. Parasitology research. 2018;117(3):801-7.

10. Falade CO, Olayemi O, Dada-Adegbola HO, Aimakhu CO, Ademowo OG, Salako LA. Prevalence of malaria at booking among antenatal clients in a secondary health care facility in Ibadan, Nigeria. African journal of reproductive health. 2008;12(2):141-52.

11. Feleke DG, Adamu A, Gebreweld A, Tesfaye M, Demisiss W, Molla G. Asymptomatic malaria infection among pregnant women attending antenatal care in malaria endemic areas of North-Shoa, Ethiopia: A cross-sectional study. Malaria Journal. 2020;19(1):1-6.

12. Francine N, Damien B, Anna F, Michael K, Christevy VJ, Felix K-K. Characterization of asymptomatic Plasmodium falciparum infection and its risk factors in pregnant women from the Republic of Congo. Acta tropica. 2016;153:111-5.

13. Gajida A, Iliyasu Z, Zoakah A. Malaria among antenatal clients attending primary health care facilities in Kano state, Nigeria. Annals of African Medicine. 2010;9(3):188-93.

14. Gibson Waweru N, Jimmy Hussein K, Oyugi EO, Omballa V, El-Busaidy H, Jeza VT. Prevalence and risk factors associated with asymptomatic Plasmodium falciparum infection and anemia among pregnant women at the first antenatal care visit: A hospital based cross-sectional study in Kwale County, Kenya. PLoS One. 2020;15(10):1-14.

15. Hillier SD, Booth M, Muhangi L, Nkurunziza P, Khihembo M, Kakande M, et al. Plasmodium falciparum and helminth coinfection in a semi urban population of pregnant women in Uganda. The Journal of infectious diseases. 2008;198(6):920-7.

16. Igwe NM, Joannes UOU, Chukwuma OB, Chukwudi OR, Oliaemeka EP, Maryrose AU, et al. Prevalence and parasite density of asymptomatic malaria parasitemia among unbooked paturients at Abakaliki, Nigeria. Journal of Basic and Clinical Reproductive Sciences. 2014;3(1):44-8.

17. Isah A, Amanabo M, Ekele B. Prevalence of malaria parasitemia amongst asymptomatic pregnant women attending a Nigerian teaching hospital. Annals of African Medicine. 2011;10(2):171-4.

18. Iwalokun BA, Iwalokun SO, Adebodun V, Balogun M. Carriage of Mutant Dihydrofolate Reductase and Dihydropteroate Synthase Genes among Plasmodium falciparum Isolates Recovered from Pregnant Women with Asymptomatic Infection in Lagos, Nigeria. Medical Principles and Practice. 2015;24(5):436-43.

19. Kattenberg JH, Tahita CM, Versteeg IAJ, Tinto H, Traoré Coulibaly M, D'Alessandro U, et al. Evaluation of antigen detection tests, microscopy, and polymerase chain reaction for diagnosis of malaria in peripheral blood in asymptomatic pregnant women in Nanoro, Burkina Faso. The American journal of tropical medicine and hygiene. 2012;87(2):251-6.

20. Kiptoo D. Factors Associated with Asymptomatic Malaria among Pregnant Women Attending Antenatal Clinic at Ridge Regional Hospital Accra, Ghana: University of Ghana; 2016.

21. Laine T. The prevalence of asymptomatic malaria and its relation to the characteristics of pregnant women in rural Malawi: Tmmpere University; 2018.

22. Maiga-Ascofare O, Rakotozandrindrainy R, Girmann M, Hahn A, Randriamampionona N, Poppert S, et al. Molecular epidemiology and seroprevalence in asymptomatic Plasmodium falciparum infections of Malagasy pregnant women in the highlands. Malaria Journal. 2015;14:1-9.

23. Martínez Pérez G, González R, Tarr Attia CK, Bardají A, Sarukhan A, Lansana DP, et al. Prevalence of plasmodium falciparum infection and antimalarial resistance among pregnant women attending antenatal care in Monrovia, Liberia. American Journal of Tropical Medicine and Hygiene. 2018;99(4):1-11.

24. Matangila JR, Lufuluabo J, Ibalanky AL, da Luz RAI, Lutumba P, Van Geertruyden JP. Asymptomatic Plasmodium falciparum infection is associated with anaemia in pregnancy and can be more cost-effectively detected by rapid diagnostic test than by microscopy in Kinshasa, Democratic Republic of the Congo. Malaria Journal. 2014;13(132):1-10.

25. Mlugu EM, Minzi O, Kamuhabwa AAR, Aklillu E. Prevalence and correlates of asymptomatic malaria and anemia on first antenatal care visit among pregnant women in Southeast, Tanzania. International Journal of Environmental Research and Public Health. 2020;17(9):1-16.

26. Nega D, Dana D, Tefera T, Eshetu T. Prevalence and predictors of asymptomatic malaria parasitemia among pregnant women in the rural surroundings of Arbaminch Town, South Ethiopia. PLoS ONE. 2015;10(4):1-11.

27. Nwaneri DU, Adeleye OA, Ande AB. Asymptomatic malaria parasitaemia using rapid diagnostic test in unbooked pregnant women in rural Ondo-south district, Nigeria. Journal of Preventive Medicine and Hygiene. 2013;54(1):49-52.

28. Nyunt M, Pisciotta J, Feldman AB, Thuma P, Scholl PF, Demirev PA, et al. Detection, of Plasmodium falciparum in pregnancy by laser desorption mass spectrometry. American Journal of Tropical Medicine and Hygiene. 2005;73(3):485-90.

29. Obebe OO, Falohun OO, Olajuyigbe OO, Lawani MA, Ajayi OA. Impact of asymptomatic plasmodium falciparum on haematological parameters of pregnant women at first antenatal visit in South-Western Nigeria. Tanzania Journal of Health Research. 2018;20(2):1-8.

30. Ogbodo SO, Nwagha UI, Okaka ANC, Ogenyi SC, Okoko RO, Nwagha TU. Malaria parasitaemia among pregnant women in a rural community Of eastern Nigeria; need for combined measures. Nigerian Journal of Physiological Sciences. 2009;24(2):95-100.

31. Ogbu GI, Aimakhu CO, Anzaku SA, Ngwan S, Ogbu DA. Prevalence of malaria parasitaemia among asymptomatic women at booking visit in a tertiary hospital, North-central Nigeria. circulation. 2015;2(29):34-6.

32. Okusanya BO, Eigbefoh JO, Ohiosimuan O, Isabu PA, Okpere EE, Inyang NJ. Utility of intradermal blood smear in the detection of asymptomatic malaria parasitaemia in pregnancy. The Nigerian postgraduate medical journal. 2009;16(3):182-5.

33. Saute F, Menendez C, Mayor A, Aponte J, Gomez-Olive X, Dgedge M, et al. Malaria in pregnancy in rural Mozambique: the role of parity, submicroscopic and multiple Plasmodium falciparum infections. Tropical medicine & international health : TM & IH. 2002;7(1):19-28.

34. Sule-Odu A, Akadri A, Adeiyi T, Sotunsa J, Durojaiye B, Oluwole A. Prevalence of malaria parasitaemia amongst asymptomatic pregnant women in Sagamu. Tropical Journal of Obstetrics and Gynaecology. 2015;32(1):118-23.

35. Zablon KN, Kakilla C, Lykina T, Minakova V, Chibago A, Zanda B. Prevalence of<i> Plasmodium falciparum</i> Malaria among Pregnant Students in Dodoma Region, Tanzania: No Cases Have Been Detected. Malaria Research and Treatment. 2015;2015(5):1-5.
