# Supplementary material for: A systematic review and meta-analysis of asymptomatic malaria infection in pregnant women in Sub-Saharan Africa: A challenge for malaria elimination efforts
Source: PLoS One. 2021 Apr 1;16(4):e0248245. doi: 10.1371/journal.pone.0248245 (PMC8016273; doi:10.1371/journal.pone.0248245)
Supplement: S1 File — (DOCX) [file pone.0248245.s003.docx]

**Study search strategies**

**Embase (129 results)**

No. Query Results

#3. #1 AND #2 122

#2. pregnan* OR pregnancy OR 'pregnant women' OR 1,189,339

antenatal OR 'prenatal care' OR prenatal

#1. 'asymptomatic parasitemia' OR 'asymptomatic 897

malaria' OR 'afebrile parasitaemia' OR

'asymptomatic malaria*' OR 'subclinical malaria'

OR 'asymptomatic plasmodium*' OR 'subpatent

malaria' OR 'plasmodium vivax asymptomatic*' OR

'plasmodium falciparum asymptomatic*' OR

'afebrile malaria'

**PubMed (78 results)**

(((((((("asymptomatic parasitemia"[Title/Abstract] OR "asymptomatic malaria"[Title/Abstract]) OR "asymptomatic malaria*"[Title/Abstract]) OR "subclinical malaria"[Title/Abstract]) OR "asymptomatic plasmodium*"[Title/Abstract]) OR "afebrile malaria"[Title/Abstract]) OR "subpatentmalaria"[Title/Abstract]) OR "plasmodium falciparum asymptomatic*"[Title/Abstract]) OR "afebrile malaria"[Title/Abstract]) AND (((((("pregnan*"[Title/Abstract] OR "pregnancy"[Title/Abstract]) OR "pregnant women"[Title/Abstract]) OR "antenatal"[Title/Abstract]) OR "prenatal care"[Title/Abstract]) OR "prenatal"[Title/Abstract]) OR ("antenata"[All Fields] AND "care"[Title/Abstract]))

**Soups (24 results)**

( TITLE-ABS-KEY ( "asymptomatic parasitemia" OR "asymptomatic malaria" OR "afebrile parasitaemia" OR "asymptomatic malaria*" OR "subclinical malaria" OR "Asymptomatic Plasmodium*" OR "afebrile malaria" OR "subjacent malaria" OR "Plasmodium vivax asymptomatic*" OR "Plasmodium falciparum asymptomatic*" OR "afebrile malaria " ) ) AND ( TITLE-ABS-KEY ( pregnan* OR pregnancy OR 'pregnant AND women'' OR antenatal OR ''prenatal AND care'' OR prenatal OR ''antenatal AND care'' ) )

**ProQuest (388 results)**

ab('asymptomatic parasitemia' OR 'asymptomatic malaria' OR 'afebrile parasitaemia' OR 'asymptomatic malaria*' OR 'subclinical malaria' OR 'asymptomatic plasmodium*' OR 'subpatent malaria' OR 'plasmodium vivax asymptomatic*' OR 'plasmodium falciparum asymptomatic*' OR 'afebrile malaria') AND ab(pregnan* OR pregnancy OR 'pregnant women' OR antenatal OR 'prenatal care' OR prenatal OR 'antenatal care')

**Web of Science (82 results)**

#2 AND #1 search result=76

Indexes=SCI-EXPANDED, SSCI, A&HCI, CPCI-S, CPCI-SSH, BKCI-S, BKCI-SSH, ESCI, CCR-EXPANDED, IC Timespan=All years

# 2 search result= 603,520

TS=(pregnan* OR pregnancy OR ''pregnant women'' OR antenatal OR '' prenatal care'' OR prenatal OR '' antenata care'')

Indexes=SCI-EXPANDED, SSCI, A&HCI, CPCI-S, CPCI-SSH, BKCI-S, BKCI-SSH, ESCI, CCR-EXPANDED, IC Timespan=All years

# 1 search result=700

TS=( "asymptomatic parasitemia" OR "asymptomatic malaria" OR "afebrile parasitaemia" OR "asymptomatic malaria*" OR "subclinical malaria" OR "Asymptomatic Plasmodium*" OR "afebrile malaria" OR "subpatent malaria" OR "Plasmodium vivax asymptomatic*" OR "Plasmodium falciparum asymptomatic*" OR "afebrile malaria ")

Indexes=SCI-EXPANDED, SSCI, A&HCI, CPCI-S, CPCI-SSH, BKCI-S, BKCI-SSH, ESCI, CCR-EXPANDED, IC Timespan=All years
