# Supplementary material for: A systematic review and meta-analysis of asymptomatic malaria infection in pregnant women in Sub-Saharan Africa: A challenge for malaria elimination efforts
Source: PLoS One. 2021 Apr 1;16(4):e0248245. doi: 10.1371/journal.pone.0248245 (PMC8016273; doi:10.1371/journal.pone.0248245)
Supplement: S2 File — (DOCX) [file pone.0248245.s004.docx]

Risk of bias assessment of included studies using a Hoy et al., 2012

| Study ID | Representation | Sampling | Random selection | Non response bias | Data collection | Case Definition | Reliability and validity of study tool | Method of data collection | Prevalence period | Numerator and denominator | Overall  Assessment |
| --- | --- | --- | --- | --- | --- | --- | --- | --- | --- | --- | --- |
| [1] | Medium risk | Low | Low | Unclear risk | Low | Low | Low | Low | Low | Low | Low |
| [2] | Low | Low | Low | Unclear | Low | Medium risk | Medium | Low | Low | Low | Moderate |
| [3] | Low | Low | Unclear | Unclear | Low | Medium | Medium | Low | Low | Low | Moderate |
| [4] | Low | Low | Low | Unclear | Low | Medium | Medium | Low | Low | Low | Moderate |
| [5] | Low | Low | Low | Unclear | Low | Low | Medium | Low | Low | Low | Low |
| [6] | Low | Low | Unclear | Unclear | Low | Low | Medium | Low | Low | Low | Moderate |
| [7] | Low | Low | Unclear | Unclear | Low | Low | Medium | Low | Low | Low | Moderate |
| [8] | Low | Low | Unclear | Low | Low | Low | Medium | Low | Low | Low | Low |
| [9] | Low | Low | Low | Unclear | Low | Low | Medium | Low | Low | Low | Low |
| [10] | Low | Low | Unclear | Unclear | Low | Low | Medium | Low | Low | Low | Moderate |
| [11] | Low | Low | Low | Unclear | Low | Low | Low | Low | Low | Low | Low |
| [12] | Low | Low | Unclear | Unclear | Low | Low | Low | Low | Low | Low | Low |
| [13] | Low | Low | Low | Unclear | Low | Low | Medium | Low | Low | Low | Low |
| [14] | Low | Low | Unclear | Unclear | Low | Low | Medium | Low | Low | Low | Moderate |
| [15] | Low | Low | Unclear | Unclear | Low | Low | Medium | Low | Low | Low | Moderate |
| [16] | Low | Low | Unclear | Unclear | Low | Low | Medium | Low | Low | Low | Moderate |
| [17] | Low | Low | Low | Unclear | Low | Low | Medium | Low | Low | Low | Low |
| [18] | Medium | Low | Low | Unclear | Low | Low | Low | Low | Low | Low | Low |
| [19] | Low | Low | Unclear | Low | Medium | Low | Low | Low | Low | Low | Low |
| [20] | Low | Low | Low | Unclear | Medium | Low | Low | Low | Low | Low | Low |
| [21] | Low | Low | Unclear | Unclear | Low | Low | Medium | Low | Low | Low | Moderate |
| [22] | Low | Low | Unclear | Unclear | Low | Medium | Low | Low | Low | Low | Moderate |
| [23] | Medium | Low | Unclear | Unclear | Low | Low | Low | Low | Low | Low | Moderate |
| [24] | Low | Low | Unclear | Unclear | Low | Low | Low | Low | Low | Low | Low |
| [25] | Low | Low | Unclear | Unclear | Low | Low | Low | Low | Low | Low | Low |
| [26] | Low | Low | Low | Low | Low | Low | Medium | Low | Low | Low | Low |
| [27] | Medium | Low | Unclear | Unclear | Low | Low | Medium | Low | Low | Low | Moderate |
| [28] | Medium | Low | Unclear | Unclear | Low | Low |  | Low | Low | Low | Moderate |
| [29] | Low | Low | Unclear |  | Low | Low | Medium | Low | Low | Low | Low |
| [30] | Low | Low | Low | Unclear | Low | Low | Medium | Low | Low | Low | Low |
| [31] | Low | Low | Unclear | Unclear | Low | Low | Medium | Low | Low | Low | Moderate |
| [32] | Medium | Low | Low | Unclear | Low | Low | Medium | Low | Low | Low | Moderate |
| [33] | Low | Low | Low | Unclear | Low | Low |  | Low | Low | Low | Low |
| [34] | Low | Low | Unclear | Unclear | Low | Low | Medium | Low | Low | Low | Moderate |
| [35] | Medium | Low | Unclear | Unclear | Low | Low | Medium | Low | Low | Low | Moderate |

1. Representation: Was the study population a close representation of the national population?

2. Sampling: Was the sampling frame a true or close representation of the target population?

3. Random selection: Was some form of random selection used to select the sample OR was a census undertaken?

4. Non-response bias: Was the likelihood of non-response bias minimal?

5. Data collection: Were data collected directly from the subjects?

6. Case definition: Was an acceptable case definition used in the study?

7. Reliability and validity of study tool: Was the study instrument that measured the parameter of interest show to have reliability and validity?

8. Data collection: Was the same mode of data collection used for all subjects?

9. Prevalence period: Was the length of the prevalence period for the parameter of interest appropriate?

10. Numerators and denominators: Were the numerator(s) and denominator(s) for the parameter of interest appropriate?

The overall risk of bias was then rated based on the number of high risk of bias per study: low (≤2), moderate (3–4), and high (≥5).

1. Adane T, Yimer M, Gelaye W, Tegegne B: **Prevalence of asymptomatic Plasmodium species infection and associated factors among pregnant women attending antenatal care at Fendeka town health facilities, Jawi District, North west Ethiopia: A cross-sectional study**. *PLoS One* 2020, **15**(4).

2. Adesina KT, Balogun OR, Babatunde AS, Sanni MA, Fadeyi A, Aderibigbe S: **Impact of malaria parasitaemia on haematologic parameters in pregnant women at booking in Ilorin, Nigeria**. *Trends in Medical Research* 2009, **4**(4):84-90.

3. Akinbo FO, Osanyinbi B, Omoregie R, Ande ABA: **Asyptomatic malaria among pregnant women in Edo State, Nigeria**. *Journal of Medicine and Biomedical Research* 2014, **13**(1):61-69.

4. Anchang-Kimbi JK, Nkweti VN, Ntonifor HN, Apinjoh TO, Tata RB, Chi HF, Achidi EA: **Plasmodium falciparum parasitaemia and malaria among pregnant women at first clinic visit in the mount Cameroon Area**. *BMC infectious diseases* 2015, **15**(1):439.

5. Anyaehie U, Nwagha UI, Aniebue PN, Nwagha TU: **The effect of free distribution of insecticide-treated nets on asymptomatic Plasmodium parasitemia in pregnant and nursing mothers in a rural Nigerian community**. *Nigerian journal of clinical practice* 2011, **14**(1):19-22.

6. Bouyou-Akotet MK, Ionete-Collard DE, Mabika-Manfoumbi M, Kendjo E, Matsiegui P-B, Mavoungou E, Kombila M: **Prevalence of Plasmodium falciparum infection in pregnant women in Gabon**. *Malaria journal* 2003, **2**(1):18.

7. Douamba Z, Bisseye C, Djigma FW, Compaoré TR, Bazie VJ, Pietra V, Nikiema JB, Simpore J: **Asymptomatic malaria correlates with anaemia in pregnant women at Ouagadougou, Burkina Faso**. *Journal of biomedicine & biotechnology* 2012, **2012**:198317.

8. Emiasegen SE, Giwa FJ, Ajumobi O, Ajayi I, Ahmed SA, Olayinka AT: **Asymptomatic Plasmodium falciparum parasitaemia among pregnant women: a health facility-based survey in Nassarawa-Eggon, Nigeria**. *Malaria World J* 2017, **15**(24):25-34.

9. Esu E, Tacoli C, Gai P, Berens-Riha N, Pritsch M, Loescher T, Meremikwu M: **Prevalence of the Pfdhfr and Pfdhps mutations among asymptomatic pregnant women in Southeast Nigeria**. *Parasitology research* 2018, **117**(3):801-807.

10. Falade CO, Olayemi O, Dada-Adegbola HO, Aimakhu CO, Ademowo OG, Salako LA: **Prevalence of malaria at booking among antenatal clients in a secondary health care facility in Ibadan, Nigeria**. *African journal of reproductive health* 2008, **12**(2):141-152.

11. Feleke DG, Adamu A, Gebreweld A, Tesfaye M, Demisiss W, Molla G: **Asymptomatic malaria infection among pregnant women attending antenatal care in malaria endemic areas of North-Shoa, Ethiopia: A cross-sectional study**. *Malaria Journal* 2020, **19**(1).

12. Francine N, Damien B, Anna F, Michael K, Christevy VJ, Felix K-K: **Characterization of asymptomatic Plasmodium falciparum infection and its risk factors in pregnant women from the Republic of Congo**. *Acta tropica* 2016, **153**:111-115.

13. Gajida AU, Iliyasu Z, Zoakah AI: **Malaria among antenatal clients attending primary health care facilities in Kano state, Nigeria**. *Annals of African medicine* 2010, **9**(3):188-193.

14. Gibson Waweru N, Jimmy Hussein K, Oyugi EO, Omballa V, El-Busaidy H, Jeza VT: **Prevalence and risk factors associated with asymptomatic Plasmodium falciparum infection and anemia among pregnant women at the first antenatal care visit: A hospital based cross-sectional study in Kwale County, Kenya**. *PLoS One* 2020, **15**(10).

15. Hillier SD, Booth M, Muhangi L, Nkurunziza P, Khihembo M, Kakande M, Sewankambo M, Kizindo R, Kizza M, Muwanga M *et al*: **Plasmodium falciparum and helminth coinfection in a semi urban population of pregnant women in Uganda**. *The Journal of infectious diseases* 2008, **198**(6):920-927.

16. Igwe NM, Joannes UOU, Chukwuma OB, Chukwudi OR, Oliaemeka EP, Maryrose AU, Joseph A: **Prevalence and parasite density of asymptomatic malaria parasitemia among unbooked paturients at Abakaliki, Nigeria**. *Journal of Basic and Clinical Reproductive Sciences* 2014, **3**(1):44-48.

17. Isah A, Amanabo M, Ekele B: **Prevalence of malaria parasitemia amongst asymptomatic pregnant women attending a Nigerian teaching hospital**. *Annals of African Medicine* 2011, **10**(2):171-174.

18. Iwalokun BA, Iwalokun SO, Adebodun V, Balogun M: **Carriage of Mutant Dihydrofolate Reductase and Dihydropteroate Synthase Genes among Plasmodium falciparum Isolates Recovered from Pregnant Women with Asymptomatic Infection in Lagos, Nigeria**. *Medical Principles and Practice* 2015, **24**(5):436-443.

19. Kattenberg JH, Tahita CM, Versteeg IAJ, Tinto H, Traoré Coulibaly M, D'Alessandro U, Schallig HDFH, Mens PF: **Evaluation of antigen detection tests, microscopy, and polymerase chain reaction for diagnosis of malaria in peripheral blood in asymptomatic pregnant women in Nanoro, Burkina Faso**. *The American journal of tropical medicine and hygiene* 2012, **87**(2):251-256.

20. Kiptoo D: **Factors Associated with Asymptomatic Malaria among Pregnant Women Attending Antenatal Clinic at Ridge Regional Hospital Accra, Ghana**. University of Ghana; 2016.

21. Laine T: **The prevalence of asymptomatic malaria and its relation to the characteristics of pregnant women in rural Malawi**. 2018.

22. Maiga-Ascofare O, Rakotozandrindrainy R, Girmann M, Hahn A, Randriamampionona N, Poppert S, May J, Schwarz NG: **Molecular epidemiology and seroprevalence in asymptomatic Plasmodium falciparum infections of Malagasy pregnant women in the highlands**. *Malaria Journal* 2015, **14**.

23. Martínez Pérez G, González R, Tarr Attia CK, Bardají A, Sarukhan A, Lansana DP, Gupta H, García-Sípido AM, Bassat Q, Aparicio AM: **Prevalence of plasmodium falciparum infection and antimalarial resistance among pregnant women attending antenatal care in Monrovia, Liberia**. *American Journal of Tropical Medicine and Hygiene* 2018, **99**(4):98.

24. Matangila JR, Lufuluabo J, Ibalanky AL, da Luz RAI, Lutumba P, Van Geertruyden JP: **Asymptomatic Plasmodium falciparum infection is associated with anaemia in pregnancy and can be more cost-effectively detected by rapid diagnostic test than by microscopy in Kinshasa, Democratic Republic of the Congo**. *Malaria Journal* 2014, **13**.

25. Mlugu EM, Minzi O, Kamuhabwa AAR, Aklillu E: **Prevalence and correlates of asymptomatic malaria and anemia on first antenatal care visit among pregnant women in Southeast, Tanzania**. *International Journal of Environmental Research and Public Health* 2020, **17**(9).

26. Nega D, Dana D, Tefera T, Eshetu T: **Prevalence and predictors of asymptomatic malaria parasitemia among pregnant women in the rural surroundings of Arbaminch Town, South Ethiopia**. *PLoS ONE* 2015, **10**(4).

27. Nwaneri DU, Adeleye OA, Ande AB: **Asymptomatic malaria parasitaemia using rapid diagnostic test in unbooked pregnant women in rural Ondo-south district, Nigeria**. *Journal of Preventive Medicine and Hygiene* 2013, **54**(1):49-52.

28. Nyunt M, Pisciotta J, Feldman AB, Thuma P, Scholl PF, Demirev PA, Lin JS, Shi L, Kumar N, Sullivan DJ, Jr.: **Detection, of Plasmodium falciparum in pregnancy by laser desorption mass spectrometry**. *American Journal of Tropical Medicine and Hygiene* 2005, **73**(3):485-490.

29. Obebe OO, Falohun OO, Olajuyigbe OO, Lawani MA, Ajayi OA: **Impact of asymptomatic plasmodium falciparum on haematological parameters of pregnant women at first antenatal visit in South-Western Nigeria**. *Tanzania Journal of Health Research* 2018, **20**(2).

30. Ogbodo SO, Nwagha UI, Okaka AN, Ogenyi SC, Okoko RO, Nwagha TU: **Malaria parasitaemia among pregnant women in a rural community of eastern Nigeria; need for combined measures**. *Nigerian journal of physiological sciences : official publication of the Physiological Society of Nigeria* 2009, **24**(2):95-100.

31. Ogbu GI, Aimakhu CO, Anzaku SA, Ngwan S, Ogbu DA: **Prevalence of malaria parasitaemia among asymptomatic women at booking visit in a tertiary hospital, North-central Nigeria**. *circulation* 2015, **2**(29):34-36.

32. Okusanya BO, Eigbefoh JO, Ohiosimuan O, Isabu PA, Okpere EE, Inyang NJ: **Utility of intradermal blood smear in the detection of asymptomatic malaria parasitaemia in pregnancy**. *The Nigerian postgraduate medical journal* 2009, **16**(3):182-185.

33. Saute F, Menendez C, Mayor A, Aponte J, Gomez-Olive X, Dgedge M, Alonso P: **Malaria in pregnancy in rural Mozambique: the role of parity, submicroscopic and multiple Plasmodium falciparum infections**. *Tropical medicine & international health : TM & IH* 2002, **7**(1):19-28.

34. Sule-Odu A, Akadri A, Adeiyi T, Sotunsa J, Durojaiye B, Oluwole A: **Prevalence of malaria parasitaemia amongst asymptomatic pregnant women in Sagamu**. *Tropical Journal of Obstetrics and Gynaecology* 2015, **32**(1):118-123.

35. Zablon KN, Kakilla C, Lykina T, Minakova V, Chibago A, Zanda B: **Prevalence of<i> Plasmodium falciparum</i> Malaria among Pregnant Students in Dodoma Region, Tanzania: No Cases Have Been Detected**. *Malaria Research and Treatment* 2015, **2015**:5.
