# Supplementary material for: A systematic review and meta-analysis of asymptomatic malaria infection in pregnant women in Sub-Saharan Africa: A challenge for malaria elimination efforts
Source: PLoS One. 2021 Apr 1;16(4):e0248245. doi: 10.1371/journal.pone.0248245 (PMC8016273; doi:10.1371/journal.pone.0248245)
Supplement: S3 File — (DOCX) [file pone.0248245.s005.docx]

Table 1. Data extraction format for the pooled odds of anemia in asymptomatic malaria-infected pregnant women compared to non-infected pregnant women in Sub-Saharan African, 2002 to 2020

| Authors | A | B | C | D |
| --- | --- | --- | --- | --- |
| Akinbo et al., 2014 | 166 | 31 | 170 | 72 |
| Douamba et al., 2012 | 41 | 82 | 8 | 70 |
| Emiasegen et al., 2017 | 36 | 90 | 19 | 97 |
| Feleke et al., 2020 | 6 | 40 | 9 | 208 |
| Francine et al., 2016 | 44 | 140 | 22 | 151 |
| Iwalokun et al., 2015 | 27 | 43 | 13 | 24 |
| Gibson et al., 2020 | 33 | 7 | 160 | 108 |
| Matangila et al., 2014 | 60 | 12 | 143 | 117 |
| Mlugu et al., 2020 | 216 | 345 | 82 | 176 |

**Key**

| Asymptomatic malaria | Anemia | |
| --- | --- | --- |
|  | Yes | No |
| Positive | A | B |
| Negative | C | D |

Table 2. Data extraction format for the pooled odds of asymptomatic malaria infection in primigravidae pregnant women compared multigravida pregnant women in Sub-Saharan African, 2002 to 2020

| Authors | A | B | C | D |
| --- | --- | --- | --- | --- |
| Anchang-Kimbi et al., 2015 | 7 | 78 | 20 | 195 |
| Emiasegen et al., 2017 | 28 | 61 | 27 | 126 |
| Feleke et al., 2020 | 3 | 75 | 12 | 173 |
| Francine et al., 2016 | 25 | 70 | 43 | 217 |
| Gibson et al., 2020 | 23 | 122 | 17 | 146 |
| Iwalokun et al., 2015 | 12 | 12 | 28 | 55 |
| Kiptoo Daniel , 2016 | 12 | 162 | 10 | 194 |
| Matangila et al., 2014 | 27 | 59 | 45 | 201 |
| Mlugu et al., 2020 | 94 | 125 | 204 | 396 |
| Nega et al., 2015 | 14 | 77 | 17 | 233 |
| Nwaneri et al., 2013 | 7 | 13 | 15 | 50 |
| Ogbodo et al., 2009 | 74 | 50 | 89 | 59 |

**Key**

| Gravidity | Asymptomatic malaria | |
| --- | --- | --- |
|  | Yes | No |
| Primigravidae | A | B |
| Multigravida | C | D |

Table 3. Data extraction format for pooled odds of asymptomatic malaria infection in first and second-trimester pregnant women compared to third-trimester pregnant women in Sub-Saharan African, 2002 to 2020

| Authors | A | B | C | D |
| --- | --- | --- | --- | --- |
| Akinbor et al., 2014 | 148 | 187 | 49 | 55 |
| Anchang-Kimbi et al., 2015 | 17 | 175 | 10 | 99 |
| Emiasegen et al., 2017 | 10 | 60 | 45 | 127 |
| Gibson et al., 2020 | 26 | 176 | 14 | 92 |
| Iwalokun et al., 2015 | 35 | 55 | 5 | 12 |
| Matangila et al., 2014 | 42 | 167 | 30 | 93 |
| Nega et al., 2015 | 21 | 202 | 10 | 108 |
| Nwaneri et al., 2013 | 14 | 26 | 8 | 37 |
| Ogbodo et al., 2009 | 99 | 64 | 27 | 82 |
| Kiptoo Daniel, 2016 | 8 | 126 | 14 | 230 |

**Key**

| Trimester | Asymptomatic malaria | |
| --- | --- | --- |
|  | Yes | No |
| First and second-trimester | A | B |
| Third-trimester | C | D |
