# Supplementary figures and images for: A systematic review and meta-analysis of asymptomatic malaria infection in pregnant women in Sub-Saharan Africa: A challenge for malaria elimination efforts
Source: PLoS One. 2021 Apr 1;16(4):e0248245. doi: 10.1371/journal.pone.0248245 (PMC8016273; doi:10.1371/journal.pone.0248245)

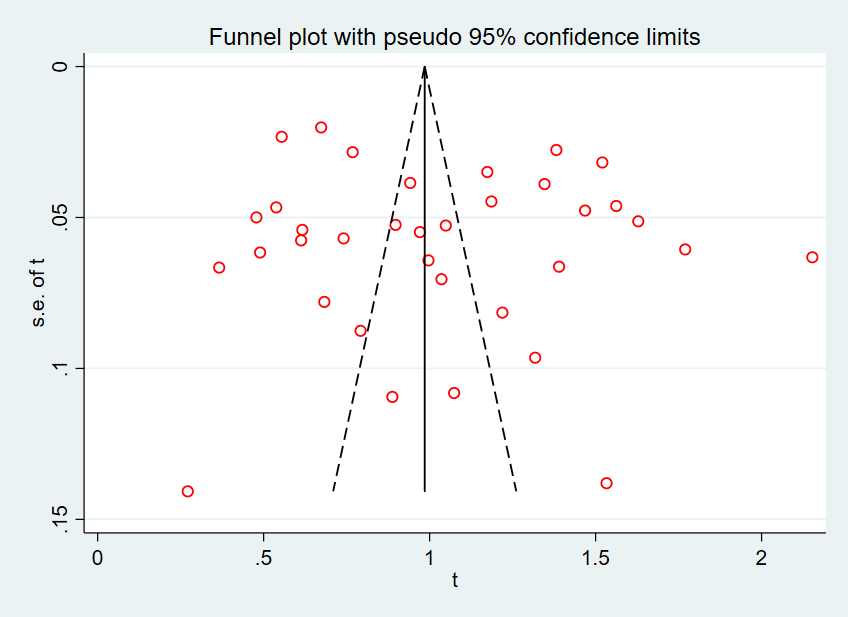

Supplement: S1 Fig — Abbreviation: se of t, standard error of t. (TIF) [file pone.0248245.s006.tif]

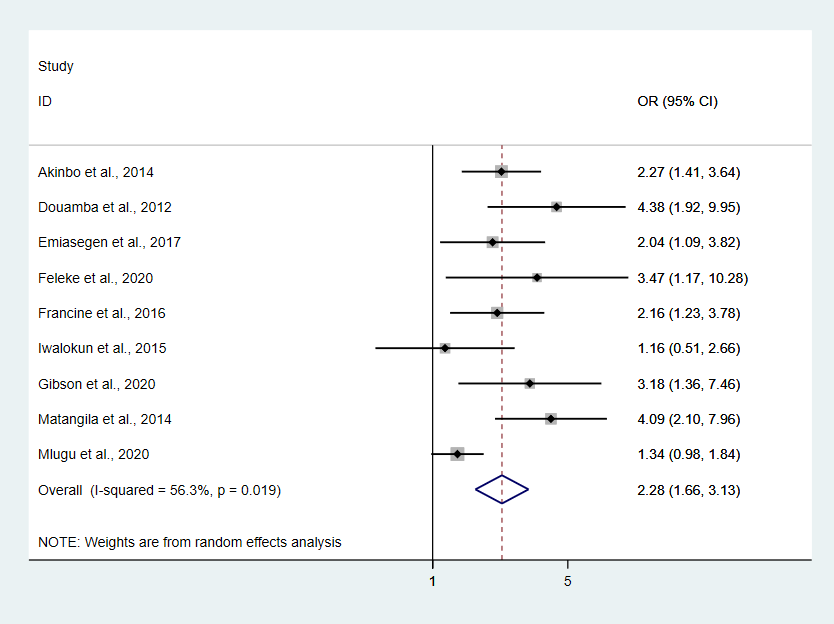

Supplement: S2 Fig — (TIF) [file pone.0248245.s007.tif]

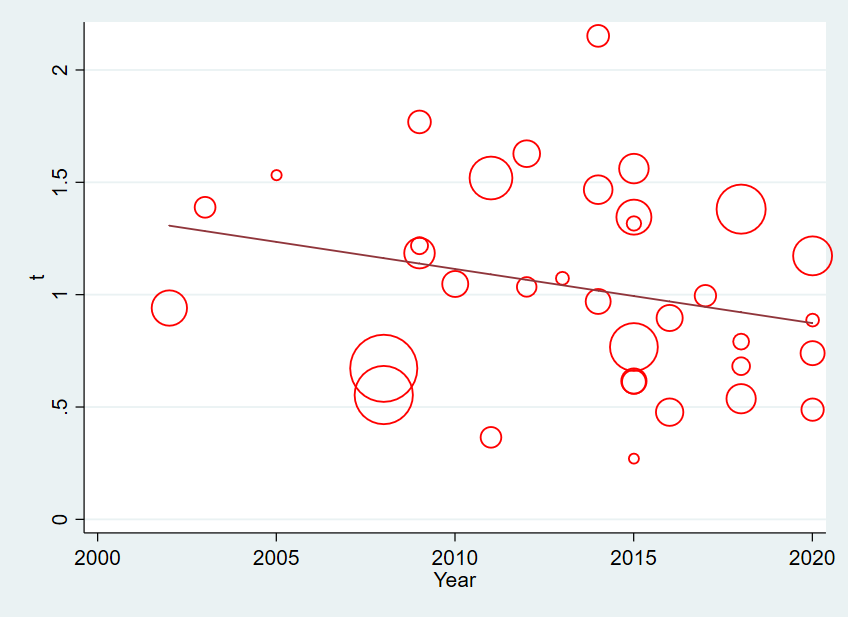

Supplement: S3 Fig — (TIF) [file pone.0248245.s008.tif]

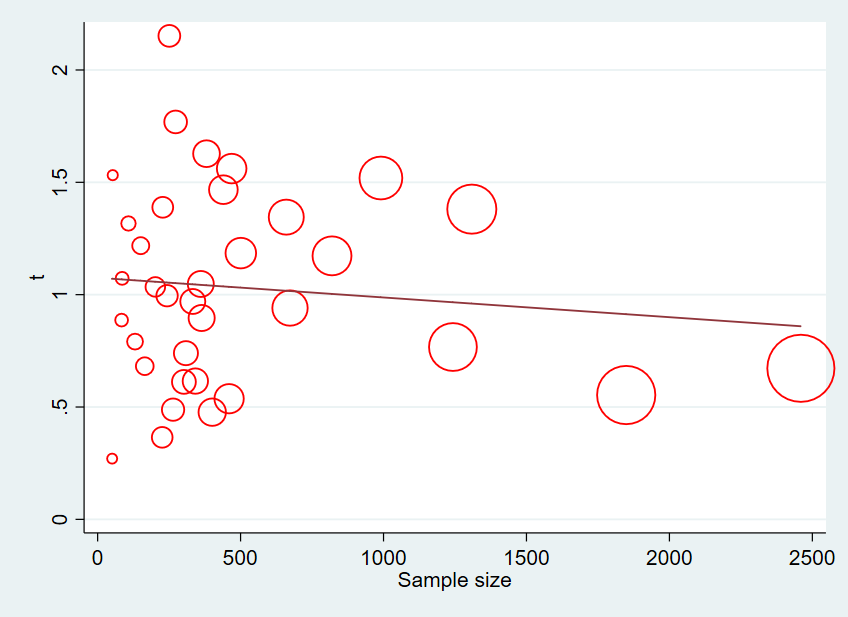

Supplement: S4 Fig — (TIF) [file pone.0248245.s009.tif]

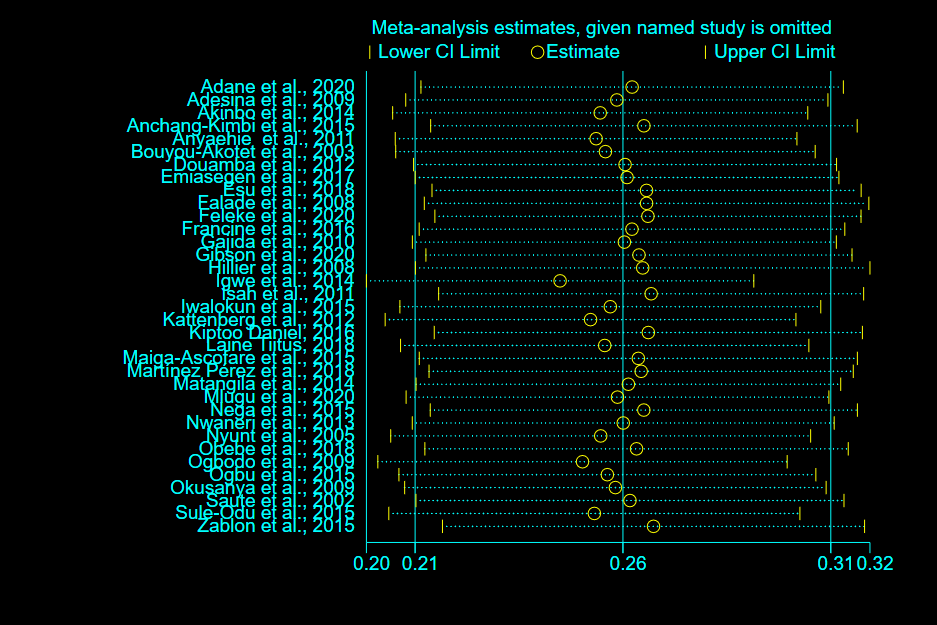

Supplement: S5 Fig — (TIF) [file pone.0248245.s010.tif]
